# Design and optimization of a high-performance multi-barrier IPMS motor for an electric scooter and bicycle

**DOI:** 10.1098/rsos.231650

**Published:** 2024-03-13

**Authors:** Halil Gör, Adem Dalcalı

**Affiliations:** ^1^ Faculty of Engineering, Department of Electrical and Electronics Engineering, Hakkari University, Hakkari 30000, Turkey; ^2^ Faculty of Engineering and Natural Sciences, Department of Electrical and Electronics Engineering, Bandırma Onyedi Eylül University, Bandırma, Balıkesir 10200, Turkey

**Keywords:** electric bicycle, e-scooter, FEM, IPMS motor, multi-barrier

## Abstract

Acknowledging the growing importance of electric vehicles (EVs) in the face of environmental concerns and increasing mobility needs, this study focuses on enhancing the performance of the electric motor, a critical component in EVs. The electric motor of these battery-powered vehicles is expected to have optimal characteristics and efficiency. This paper presents a comprehensive investigation into the design and optimization of a multi-barrier interior permanent magnet synchronous motor tailored for e-scooters and electric bicycles. A multi-barrier rotor structure is proposed and analysed through finite element method simulations to optimize key design parameters. The parametric analysis examines the influence of geometric variables on key motor performance criteria, including efficiency, cogging torque and weight. A correlation analysis of the variables was conducted. A very high positive correlation (0.999) was revealed, especially between the ‘magnet duct dimension parameter’ and efficiency. A very high negative correlation (−1) was found between ‘distance from duct bottom to shaft surface’ and efficiency. The optimized motor design achieves a theoretical improvement of around 7% in efficiency, reaching an overall efficiency of 89.86%. This study highlights the consideration of multiple factors such as efficiency, cogging torque and weight in the design process for the development of sustainable and high-performance EV motor designs.

## 1. Introduction

### 1.1. Background, definition and motivation

The development of electric vehicles (EVs) and vehicles with internal combustion engines began simultaneously. However, the battery problem in EVs and the earlier start of the mass production of vehicles with internal combustion engines have caused EVs to remain in the background. The environmental concerns and oil crises, especially in recent years, have brought the use of EVs as an alternative to the agenda. The increase in mobility in the world has also encouraged the development of alternative means of transportation. The use of e-scooters and electric bicycles has become very popular, especially in cities with heavy traffic. The performance of EVs is directly associated with the selection of the electric motor used. The electric motor to be designed for EVs is expected to provide high efficiency, high power/torque density, low torque fluctuation and low cost. These goals can be achieved by improvements made in the rotor, stator or winding design. Magnet, core and structural materials, geometry or layout, winding configuration and the number and structure of slots can be considered variables.

### 1.2. Related works

In parallel with the developments in the fields of materials and electronics, there are increasing numbers of studies on EVs [1–6]. In particular, the developments in battery technology have accelerated these studies and made them more compelling. The right choice of the motor is critically important since it provides the EV with the needed torque. DC motors, asynchronous motors, synchronous motors and switched reluctance motors are used in EVs [7,8]. Asynchronous motors fall behind permanent magnet machines in power density, although they are easy to manufacture and require less maintenance [9,10]. Although high efficiency can be provided by asynchronous motors designed for EVs, they require complex control [11]. Due to the presence of brushes and collectors in conventional DC machines, there is usually a need for maintenance [12]. Thus, switched reluctance motors are preferred, particularly in scooters and smaller-sized EVs, as the absence of windings and magnets in the rotor leads to the formation of a solid structure [13]. However, due to its nature, it leads to torque fluctuation and acoustic noise, which adversely affects users’ experience. Furthermore, these motors must have position sensing [14,15]. Permanent magnet synchronous machines are preferred in industrial applications as they provide high power density, high efficiency and low volume and weight. In the literature, there are studies on permanent magnet synchronous motors (PMSMs) with different structures for EVs [16].

The performance of permanent magnet machines is closely associated with the quality of air gap flux distribution. The geometry of the stator slots and rotor flux barriers affect the air gap flux distribution. Another parameter affecting the flux distribution is the structure of magnets and rotor topology [17,18]. Regarding the rotor structure, PMSMs can be classified as surface-mounted, surface-embedded and internal magnet [19]. Interior permanent magnet synchronous (IPMS) motors used in EVs are equipped with different rotor structures (spoke-type PMs, tangential-type PMs, *U*-shape PMs and *V*-shape PMs) [20]. IPMS machines are commonly used in the EV industry due to their mechanical strength for high-speed applications [21,22]. Permanent magnet synchronous machines have equal inductance in the *d–q* axis in surface-mounted structures. In an IPMS machine, where permanent magnets are embedded in the rotor, the inductance of the *q*-axis is greater than the *d*-axis [23–26]. Upon examining studies in the literature, a new asymmetric permanent magnet rotor structure was proposed in the study to reduce torque fluctuation. The electromagnetic analysis determined that the asymmetric structure reduced the torque fluctuation compared to the conventional *V*-shaped IPM machine [27]. In a similar study, an inner rotor *V*-shaped IPM vernier motor was proposed as an alternative to *V*-shaped IPM machines, which are commonly used in hybrid EVs. Although the volume of the proposed motor was 35% smaller compared to the *V*-shaped IPM, it provided the same output torque. In the study, the torque value was increased using multi-objective design optimization. Furthermore, an improvement of 44% was achieved by optimizing the torque value by the genetic algorithm method [28]. Torque fluctuation is an important parameter affecting the vehicle’s driving comfort. In the study conducted to reduce torque fluctuation, a multi-barrier rotor geometry was adopted, and the air gap flux density waveform was aimed to be in a sinusoidal form. To this end, a general analytical model applicable to different rotor flux barriers and poles was suggested [29]. A permanent magnet IPM motor with an oblique *V* structure was proposed to improve the torque, and it was ensured that the maximum torque was produced with this structure. It was observed that the structure with the outer flux barrier had higher average torque compared to the one with the inner flux barrier [30]. Another important factor affecting torque fluctuation is the cogging torque in permanent magnet machines. These effects can be eliminated or reduced by conducting studies on the position or geometry of magnets [31].

### 1.3. Contribution of the paper

In EV applications, the efficiency, weight and performance of the vehicle depend on the characteristics of the electric motor used. Therefore, a motor designed for EVs is expected to consider many criteria, such as efficiency, torque fluctuation, cogging torque and weight. High cost is a problem, particularly in electrical machines using rare earth magnets. Thus, the main goals of machine designers are to increase torque density and efficiency in electrical machines and reduce production costs. The motors of electric bicycles and e-scooters, frequently studied in the literature, are in the range of 200–750 W [32–34]. For this purpose, a 400 W multi-barrier IPMS motor was designed for e-scooters and bicycles. The optimization of IPMS was performed using FEM. The main contributions of this study are summarized as follows.

— The level and direction of the relationship between design variables and performance have been determined using correlation analysis.
— The correlation analysis serves as a guide for designers, helping them prioritize and focus on critical variables that significantly impact motor performance.
— The motor efficiency value, which is critically important for battery-powered EVs, has been optimized at the design stage.

## 2. Specification of the designed multi-barrier interior permanent magnet motor

Magnets can be placed on the surface or embedded in permanent magnet machines. The use of IPM machines in EVs is increasing continuously since they provide wide speed control, low weight, high power and efficiency. In the literature, different rotor structures of IPM motors are proposed. These structures can be classified as *V*-shaped, double magnet shape, delta shape, hybrid delta shape, hybrid double *V-*shape, rectangular single pole and multi-barrier magnet [35,36].

The design of electric motors can be initiated with the sizing equation given in equation (2.1) [37]:


(2.1) $$S=11.K_{w1}.\bar{B}.ac.{\left(\frac{D}{1000}\right)}^{2}.\left(\frac{L}{1000}\right).\left(n\right),$$

where S is the apparent power as VA, ac is the specific electrical loading as A/m, $\bar{B}$ is the specific magnetic loading in Tesla, D and L are the stator bore diameter and length in mm, respectively, n is the rated speed in rps. $K_{w1}$ is the winding factor and is expressed in equation (2.2) with the distribution coefficient ( $k_{d}$ ) and the step coefficient ( $k_{p}$ ) [37].


(2.2) $$K_{w1}=k_{d}.k_{p}.$$

The copper loss in the conductor is effective while determining the value of the specific electrical load. Therefore, the specific electrical loading is limited by the temperature increase that will occur in the machine. The specific magnetic loading is limited by the maximum flux density in the core. The electrical input power of the IPM machine can be expressed by equation (2.3) [38]:


(2.3) $$P_{in}=\frac{3}{2}\left(V_{ds}i_{ds}+V_{qs}i_{qs}\right),$$

where $V_{ds}$ is the terminal voltage of the *d*-axis, $V_{qs}$ is the *q*-axis terminal voltage in V, $i_{ds}$ is the *d*-axis current in A and $i_{qs}$ is the *q*-axis current in A. In these machines, copper loss and iron loss can be calculated by equations (2.4 and 2.5), respectively [38]:


(2.4) $$P_{copper}=R_{a}\left(I_{d}^{2}+I_{q}^{2}\right),$$

(2.5) $$P_{iron}=\frac{V_{od}^{2}+V_{oq}^{2}}{R_{c}}.$$

$I_{d}$ and $I_{s}$ are the *d*-axis and *q*-axis current, respectively, $R_{a}$ is the phase resistance of the armature winding, $V_{od}$ and $V_{oq}$ are the *d*-axis and *q*-axis induced voltage, respectively, and $R_{c}$ is the iron loss resistance. Denoting friction and wind losses as $P_{W\&F}$ , total loss is then expressed as [38]


(2.6) $$P_{loss}=P_{copper}+P_{iron}+P_{W\&F}.$$

Accordingly, the efficiency of the machine can be calculated by equation (2.7) [37,38]:


(2.7) $$\eta =\frac{P_{in}-P_{loss}}{P_{in}}.$$

The design parameters of the IPM machine discussed in the study are presented in table 1.

**Table 1.** Properties of the motor.

**parameter**	**value**	**parameter**	**value**
number of poles	4	rotor inner diameter (mm)	26
stator outer diameter (mm)	118	steel type	M36
stator inner diameter (mm)	75	magnet type	N35
length (mm)	56	magnet width (mm)	28
number of slots	24	magnet thickness (mm)	5
rotor outer diameter (mm)	74	coil pitch	5

**Figure 1.**
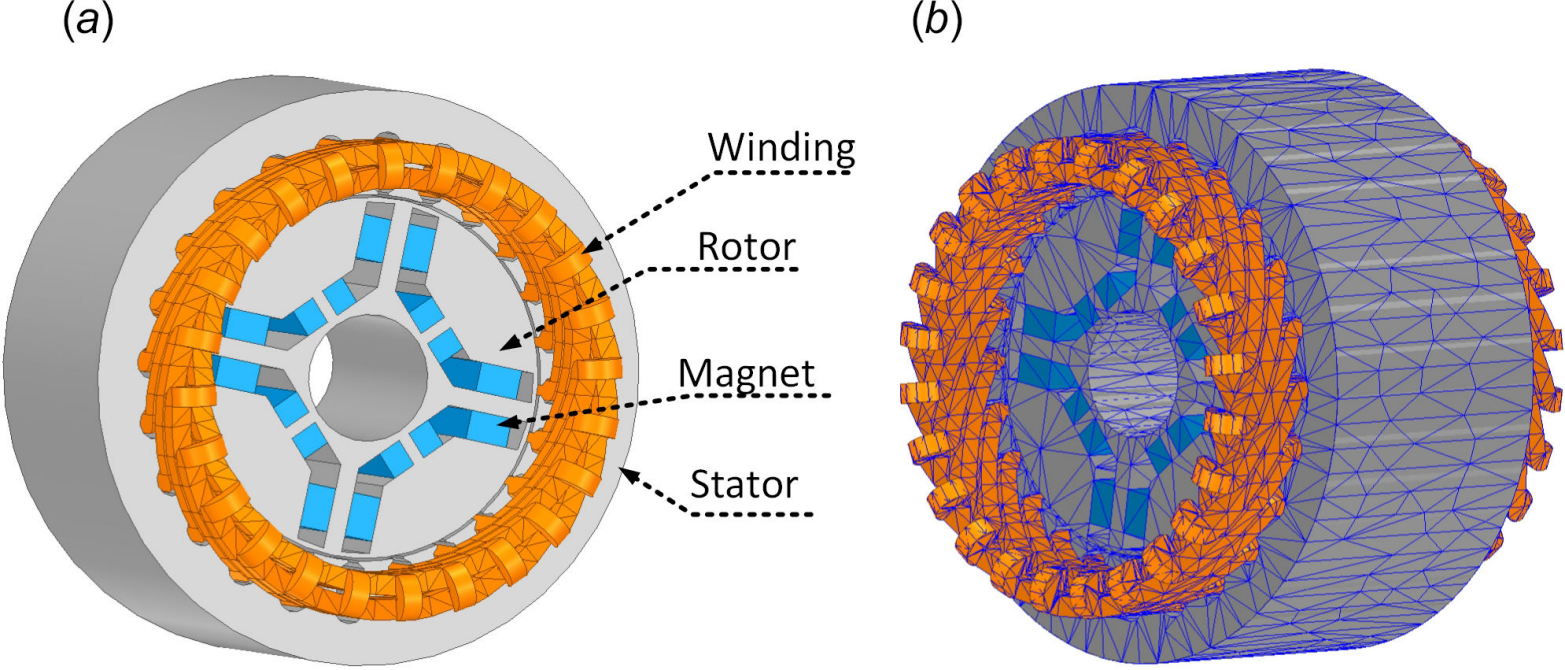
Three-dimensional model of the initial design (*a*) and mesh structure (*b*).

The three-dimensional view and mesh structure of the motor of the above characteristics are presented in figure 1.

## 3. Parametric analysis method

While designing electrical machines, it is crucial to consider mechanical and thermal constraints and the material effect while performing analyses. The designer can reach the solution of these problems after long analysis, taking into account restrictions such as cost, space and weight. The use of mathematical-based programs in solving such problems provides the designer with an advantage in terms of time and cost. The finite element method (FEM) is used in the design and analysis of electrical machines [39]. In this method, the solution of quantities that can be expressed with partial differential equations and are continuous in a certain region can be determined. The model is based on examining structures called meshes, which are divided into a finite number of regions. The number of meshes directly affects the accuracy of the analysis. With the application of FEM, important design parameters of the machine, such as induced torque, magnetic flux density, efficiency and power loss, are determined with very high accuracy [40].

First, the motor was designed using sizing equations. The motor structure was constructed in ANSYS EM/RMxprt based on the theoretical calculations. Magnet, winding and core materials were selected from the RMxprt library. Then the FEM analysis of the motor was obtained and verified via ANSYS EM Suite 19.2. The mesh structure of the motor was determined automatically in the program. A basic mesh with surface approximation settings is generated by ANSYS EM. Afterwards, the software automatically increases the number of meshes, especially in corners and air gaps. Vector-potential boundary conditions were used for the models. Transient (0–2 ms) and parametric analyses were used to compute and improve electromagnetic outputs such as magnetic flux density. Magnetic flux distributions in the motor were observed with magnetostatic analyses. Critical areas of the core were examined with magnetostatic analysis at the rated load and no-load conditions of the motor.

The effects of geometric parameters, which significantly affect motor performance, are examined in this section. The optimum values of the design parameters determined by the analyses were obtained. In the parametric solution method, determining the lower and upper limits and the solution range allows us to examine the effect of the studied parameter. By keeping the other design parameters constant, the analysis is performed by changing only the parameter that is desired to be investigated. At this point, increasing the sensitivity increases the accuracy and extends the solution time [41]. The limits of the variables of the designed motor’s rotor are defined in table 2.

**Table 2.** Parameters and limits of the rotor.

**parameter**	**min value (mm)**	**max value (mm)**	**step (mm)**
flat-bottom duct, *X*_1_	0.5	4	0.5
distance from duct bottom to shaft surface, *X*_2_	1	5	1
magnet duct dimension, *X*_3_	0.5	4	0.5
magnet thickness, *X*_4_	2	6	0.5

**Figure 2.**
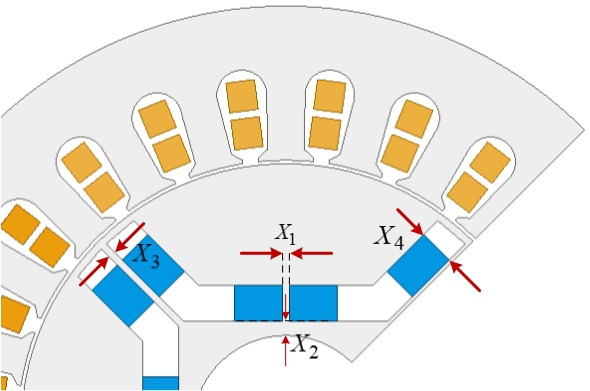
The sectional view of the motor and the variables.

Figure 2 shows the representation of the determined variables on the rotor.

## 4. Correlation and comparative analysis

**Figure 3.**
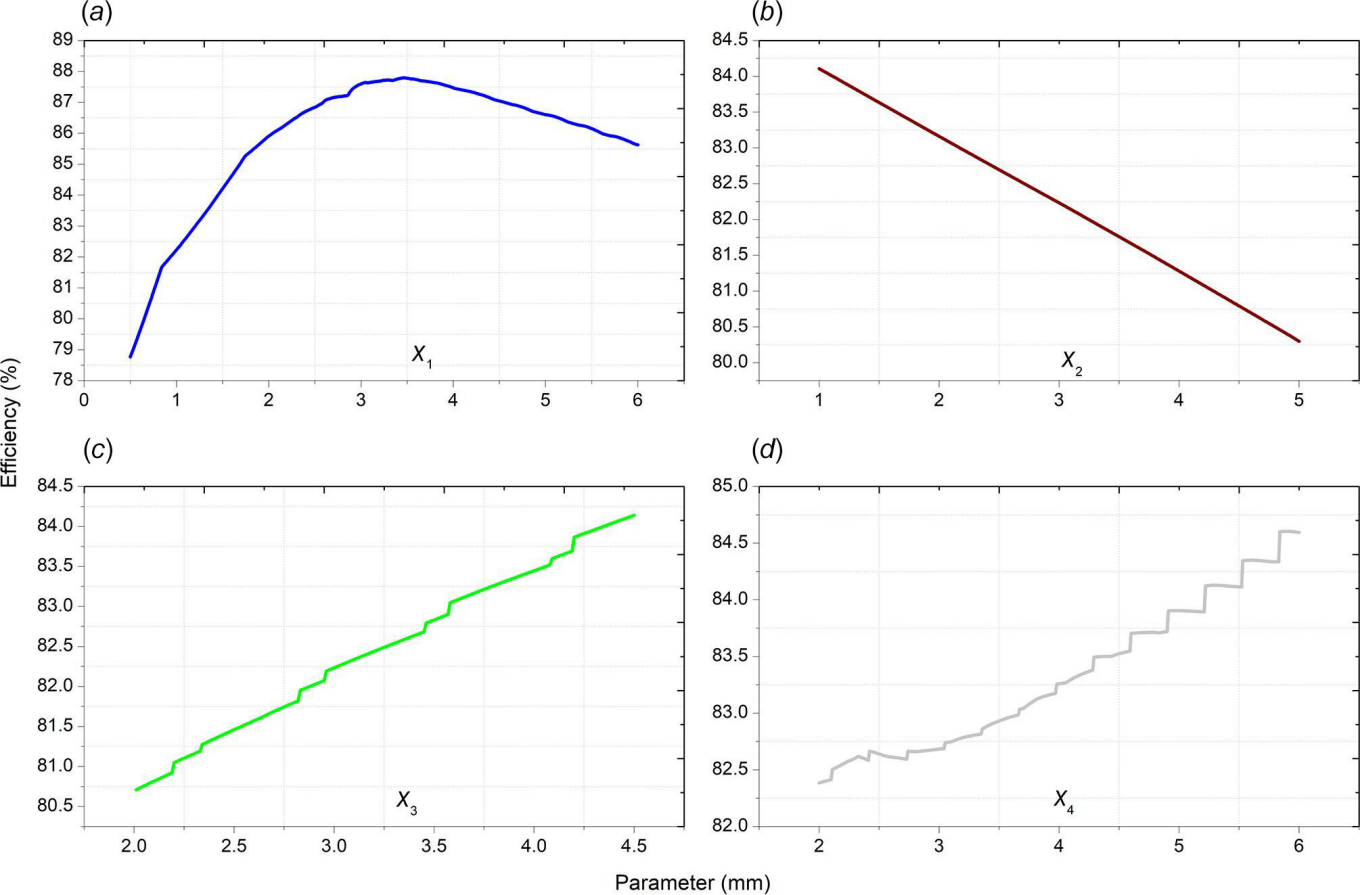
(*a*–*d*) Efficiency values used in correlation analysis.

In this section of the study, the relationship between the analysed parameters of the motor and the motor efficiency is investigated. Hence, correlation analysis was conducted. The data used in the correlation analysis are graphically presented in figure 3. Spearman correlation analysis was conducted using the SPSS v.22 program to determine the linear relationship between variables.

The degree and direction of the relationship between the variables are determined by correlation analysis. In a negative relationship, one of the variables increases while the other decreases. However, in a positive relationship, variables increase or decrease simultaneously. The correlation value is zero when there is no relationship between them. Upon examining the level of relationship between the variables, 0–0.19 can be classified as a very low correlation, 0.2–0.39 can be classified as a low correlation, 0.4–0.59 can be classified as a moderate correlation, 0.6–0.79 can be classified as a high correlation and 0.8–1 can be classified as a very high correlation [42]. Table 3 contains the effect levels and direction of the variables.

**Table 3.** Relationship of the variables.

	***X*_1_**	***X*_2_**	***X*_3_**	***X*_4_**	**efficiency**
*X*_1_	1	0	0	0	0.636
*X*_2_	0	1	0	0	−1
*X*_3_	0	0	1	0	0.999
*X*_4_	0	0	0	1	0.985
efficiency	0.636	−1	0.999	0.985	1

According to the correlation analysis results, the highest correlation was found to be between *X*_3_ and efficiency, which can be classified as a very high positive correlation. Likewise, there was a positive and very high correlation with *X*_4_. Another positive correlation was obtained with *X*_1_ as a high correlation. On the other hand, there was a negative and very high correlation between *X*_2_ and efficiency. Therefore, to increase efficiency, it is necessary to particularly focus on parameters *X*_3_ and *X*_4_ in the later studies.

**Figure 4.**
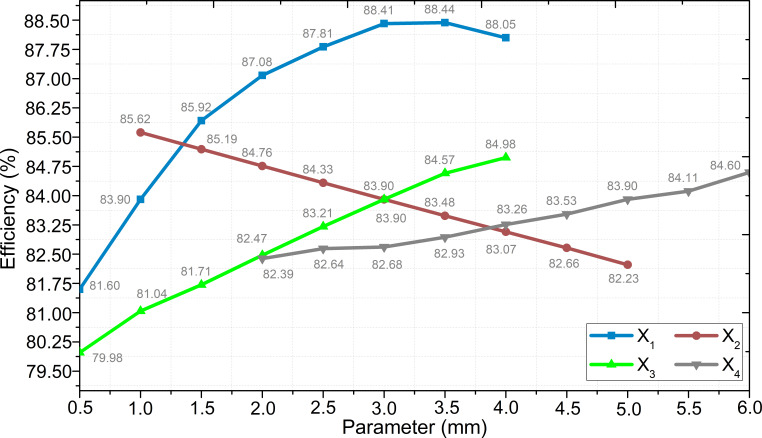
Effect of the variables on motor efficiency.

The study investigated the effect of the four variables selected in the rotor part on the motor efficiency at the specified limits and precision. A total of 5184 different structures were created with the determined variables, and it was aimed to obtain the best efficiency value. All machine parameters were taken as constant, except for the specified variable. Figure 4 shows the effect of the parameters on efficiency.

**Figure 5.**
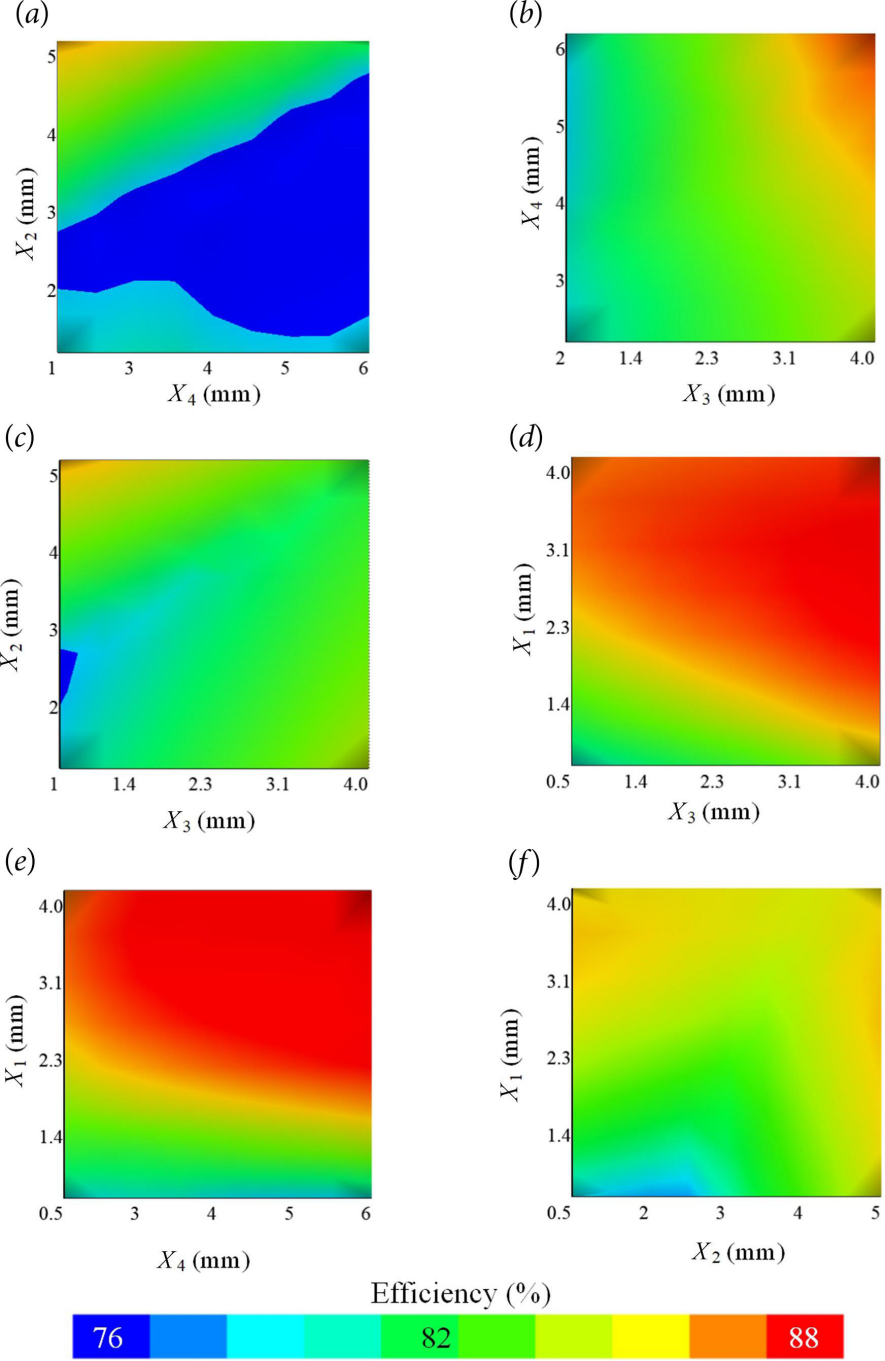
The combined effect of the parameters on efficiency (*a*–*f*).

The *X*_1_ variable has been increased in 0.5 mm increments within the range of 0.5–4 mm, and the efficiency value reached its peak at 3.5 mm, with a percentage of 88.44%, followed by a decrease. The lowest efficiency for *X*_1_ was determined to be 81.59% at 0.5 mm. The distance value from the magnet to the shaft, represented by *X*_2_, was inversely proportional to the efficiency. The highest efficiency, reaching 85.62%, occurred when the distance value was 1 mm. The lowest efficiency value for this parameter was determined to be 82.23% at the highest value of *X*_2_, which is 5 mm. When the *X*_3_ variable increased in 0.5 mm increments within the range of 0.5–4 mm, it was observed that the efficiency increased linearly. The highest efficiency, at 4 mm value of *X*_3_, was 84.98%, while the lowest efficiency, at 0.5 mm value of *X*_3_, was recorded as 79.98%. Finally, upon examining the change in efficiency and the magnet thickness and magnet duct dimension, it was observed that the efficiency was affected relatively less than other parameters. The efficiency value increased from 82.39% to 84.60% when *X*_4_ was increased from 2 to 6 mm. After obtaining the effect of each parameter separately, the effect of the parameters on efficiency together was investigated. The variation of the efficiency with the variables is presented in figure 5.

Upon evaluating *X*_2_ and *X*_4_ together, the highest efficiency value of 84.3% was found when *X*_4_ was 2 mm and *X*_2_ was 5 mm. Upon evaluating *X*_2_ and *X*_3_ together, the highest efficiency value of 84.3% was obtained when *X*_2_ was 5 mm and *X*_3_ was 0.5 mm. Finally, when the effect of *X*_2_ on efficiency with *X*_1_ was analysed, the highest efficiency was 85.1% when *X*_1_ was 2.7 mm and *X*_2_ was 5 mm. In the binary analysis, the highest efficiency value acquired was 87.4% when *X*_4_ was 6 mm and *X*_1_ was 2.95 mm. The design variables that provided the best efficiency value and the initial design variables are presented in table 4.

**Table 4.** Initial design and parameters of the optimized design.

**parameter**	**initial design**	**optimized design**
*X*_1_ (mm)	1	2.5
*X*_2_ (mm)	3	1.5
*X*_3_ (mm)	3	4
*X*_4_ (mm)	5	6

It is important to know the magnetic field distribution when calculating the working magnifications. In electrical machines, magnetic fields are expressed by Maxwell’s equations [43].


(4.1) $$\nabla \times \vec{H}=\vec{J},$$

(4.2) $$\nabla \times \vec{E}=\frac{-\partial B}{\partial t}.$$

In the equation, $\vec{H}$ refers to the magnetic field strength in A/m, $\vec{J}$ refers to the current density in A/mm^2^, $\vec{E}$ refers to the electric field intensity in N/C and $\vec{B}$ refers to the flux density in T. Magnetic vector potential in terms of magnetic flux density is expressed as [43]


(4.3) $$\vec{B}=\nabla \times \vec{A}.$$

The basic formulation of the vector potential for the magnetic field is expressed by equation (4.4) [43]:


(4.4) $$\nabla \times \left(v\nabla \times \vec{A}\right)=\vec{J}.$$

In the equation, v is variable due to the nonlinearity of magnetic flux density and is expressed as follows [43]:


(4.5) $$v=\frac{\partial B}{\partial H}.$$

The distribution of the magnetic flux density can be calculated by equation (4.6) for two-dimensional analysis and equation (4.7) for three-dimensional analysis [43].


(4.6) $$\frac{\partial }{\partial x}\left(v\frac{\partial A}{\partial x}\right)+\frac{\partial }{\partial y}\left(v\frac{\partial A}{\partial y}\right)=-\vec{J},$$

(4.7) $$\frac{\partial }{\partial x}\left(v\frac{\partial A}{\partial x}\right)+\frac{\partial }{\partial y}\left(v\frac{\partial A}{\partial y}\right)+\frac{\partial }{\partial z}\left(v\frac{\partial A}{\partial z}\right)=-\vec{J}.$$

**Figure 6.**
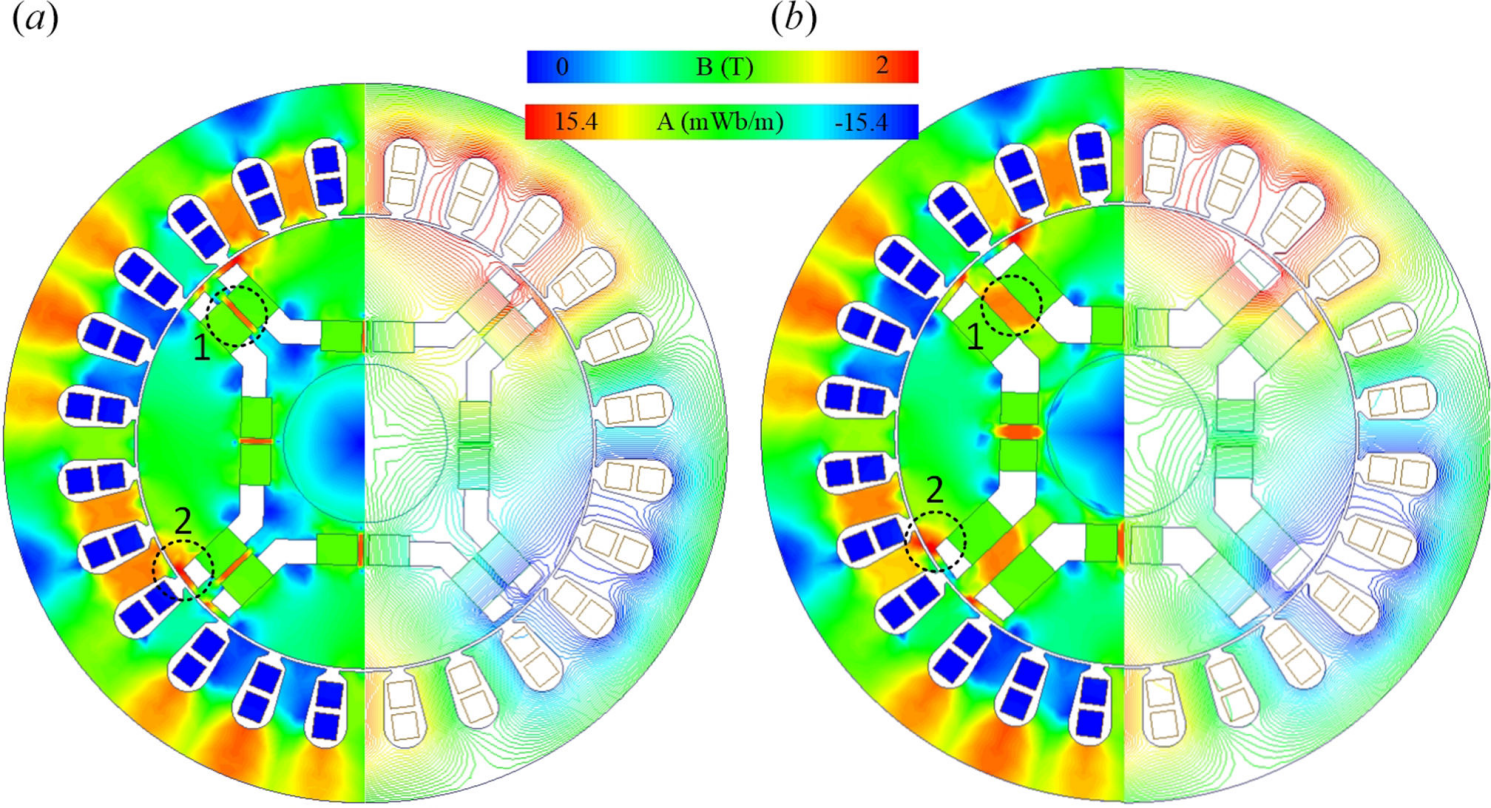
Distribution of magnetic flux density, (*a*) initial design, (*b*) optimized design.

The distribution of magnetic flux density and flux path obtained in the *x*–*y* section of the initial and optimum designs of the motor are presented in figure 6.

Upon analysing the flux distribution in the optimized design, a flux distribution similar to that of the initial design was obtained since no structural changes were made in the stator part. The core material used is M36, and a proper flux distribution was achieved. In the region marked (1) in figure 6, since *X*_3_ was increased in the optimized design of the rotor part, the flux path expanded and the flux density decreased relatively. While the rotor yoke flux density was 0.6206 T in the initial design, it decreased to 0.3972 T in the optimized design. The flux density at the point (−30, −20 and 0) in region number 2 was 1.8506 T in the initial design, while it became 1.3617 T in the optimized design. This reduction could contribute to improvements in motor efficiency and performance, as lower flux density values may lead to reduced losses.

**Figure 7.**
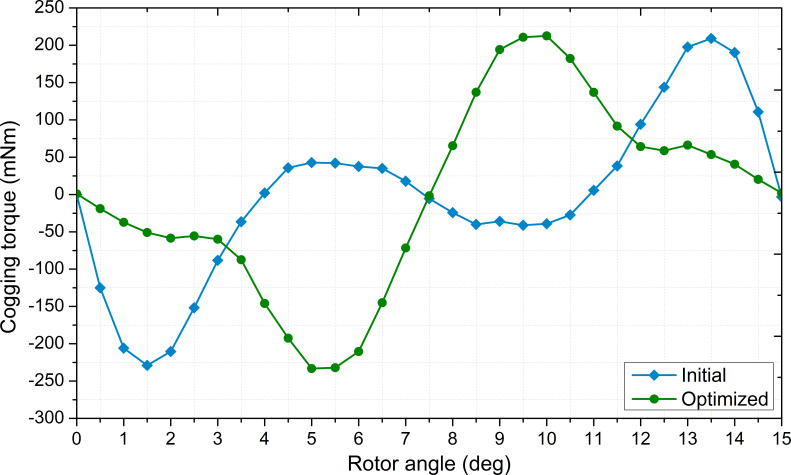
Variation of the cogging torque with rotor position.

Cogging torque is another important parameter in permanent magnet machines. In permanent magnet machines, the cogging torque occurs due to the interaction between the stator teeth and the magnets when there is no excitation in the windings. Cogging torque leads to acoustic noise and vibration in the machine [44,45]. This torque is associated with the geometric structure of the machine. Thus, cogging torque is affected by structural variables such as magnet geometry, stator slot number and structure and pole number [46,47]. Cogging torque is expressed in terms of rotor position and air gap flux by equation (4.8) in Nm [48]:


(4.8) $$T_{cog}=\frac{1}{2}\left(\emptyset_{air}^{2}\right).\left(\frac{dR}{d\theta }\right),$$

where the air gap flux quantity is expressed as $\emptyset_{air}$ (Wb) and the reluctance is expressed as R (1 /H). The position of the rotor is given as θ . As evident from the equation, the cogging torque value is a function of the air gap reluctance. Therefore, a structural change in the air gap also affects the cogging torque. The air gap change of the motor is periodic. Consequently, the value of the cogging torque also changes periodically. The periodicity of the cogging torque ensures that this value can be expressed as a Fourier series in equation (4.9) [49]:


(4.9) $$T_{cog}=\sum_{k=1}^{\infty }T_{mk}sin\left(mk\theta \right),$$

where the Fourier coefficient is $T_{mk}$ , m is the least common multiple of the number of stator slots and the number of rotor poles. In the finite element analysis performed to determine the cogging torque, it was obtained by changing the rotor position with 0.5° increments without excitation to the windings. Figure 7 presents the variation of the cogging torque. In electrical machines, many performance parameters are interrelated such that, in some cases, improvements in efficiency can negatively impact another performance criterion. In this study, nevertheless, while efficiency was improved, cogging torque was not adversely affected and remained nearly constant.

Since EVs are powered by batteries, motor is key to the overall efficiency of the vehicle. Therefore, the improvement of motor efficiency is the focus of the current study. The performance of the motors is presented in table 5. The efficiency was improved by approximately 7% compared to the initial design. Cogging torque and rated torque remained approximately the same as efficiency increased.

**Table 5.** Performance of the initial design and the optimized design.

**parameter**	**initial design**	**optimized design**
efficiency (%)	82.23	89.86
torque (Nm)	2.545	2.540
cogging torque (mNm)	229.05	232.07

Moreover, when evaluated in terms of weight, in the initial design, the stator was 2.77 kg, the rotor was 1.37 kg, the total magnets were 0.24 kg and the total copper was 0.82 kg, resulting in a total motor weight of 5.2 kg. In the optimized design, the stator remains at 2.77 kg, the rotor was 1.32 kg, the total magnets were 0.27 kg and the total copper was 0.82 kg, resulting in a slightly lower motor weight of 5.18 kg.

In IPM machines, magnet shape and rotor structure have an impact on the efficiency, torque ripple and rated torque [50,51]. Rotor geometric variables were changed within certain limits and average torque and other performance parameters were discussed by Yang *et al*. [52]. The efficiency of the machine can be optimized by geometric changes in the rotor and magnet [52–54]. A similar effect was achieved for the IPM machine by changing the geometric parameters of both the rotor and the stator [55,56]. In this study, similar to the literature, efficiency optimization was carried out with four variables defined in the rotor section. In addition to theoretically increasing the efficiency by around 7%, no negative impact was observed on cogging torque, rated torque and total engine weight.

## 5. Conclusion

Electric bicycles and scooters are considered in many instances to be reliable and eco-friendly alternatives to vehicles with internal combustion engines. This study addresses the crucial aspects of designing and optimizing an IPMS motor specifically tailored for electric scooters and bicycles. Key design parameters were optimized through the implementation of a multi-barrier rotor structure and a comprehensive parametric analysis utilizing FEM simulations. Correlations between geometric variables determined in the rotor and motor efficiency were noted. The efficiency of the motor was improved from 82.23% to 89.86%, while the cogging torque and the rated torque remained approximately constant. The cogging torque was 229.05 mNm in the initial design and 232.07 mNm in the optimized design. In addition, motor weight was slightly reduced from 5.2 to 5.18 kg. In the correlation analysis, it was determined that magnet duct dimension (*X*_3_) was the parameter that had the most positive impact on efficiency. Also, the distance from the duct bottom to the shaft surface (*X*_2_) parameter was the most important parameter that negatively affected the efficiency.

In future research, the cost factor can be examined when optimizing motor efficiency. The parameters of the motor can be determined by the optimization algorithms, regardless of the designer’s experience. Similar to the physical changes made in the rotor part, the effect of the stator slot can also be examined. The study’s outcomes provide valuable insights for future advancements in electric motor design, supporting the ongoing transition towards more environmentally friendly and high-performance EVs.

## Data Availability

Data available from the Dryad Digital Repository [57].
